# Benthic macroinvertebrates as reference indicators for monitoring of anthropogenic isotope ^137^Cs contamination in the marine environment

**DOI:** 10.1007/s11356-021-16538-y

**Published:** 2021-10-01

**Authors:** Michał Saniewski, Tamara Zalewska, Wojciech Kraśniewski

**Affiliations:** Institute of Meteorology and Water Management, National Research Institute, Waszyngtona 42, 81–342 Gdynia, Poland

**Keywords:** ^137^Cs,, Indicators,, Benthic macroinvertebrates,, Baltic Sea

## Abstract

Due to the limited studies dealing with isotopes levels in benthic macroinvertebrates other than *Mytilus* sp. in period 2011–2018, macroinvertebrates and seawater samples from the southern Baltic Sea were collected. Activity of ^137^Cs was measured in most common benthic macroinvertebrates inhabiting southern Baltic Sea in the context of choosing the best bioindicators. Taking into account the narrow range of CR coefficients, statistically significant correlation between ^137^Cs activities in seawater and species, a high frequency of occurrence and thus high biomass and the common occurrence, only *Limecola balthica* could potentially serve as indicators for shallow and deep-sea areas accordingly. Widely spread *Mytillus trossulus* was characterised by large size variability in samples and shows no significant correlation between activities in species and seawater and appears not so common as *L. balthica*.

**Supplementary Information:**

The online version contains supplementary material available at 10.1007/s11356-021-16538-y.

## Introduction

Radiocesium (^137^Cs) is an anthropogenic radioactive isotope produced by nuclear fission with a physical half-life of 30 years. Due to the chemical properties similar to potassium and accumulation by living organisms, it is considered as one of the most harmful of long-lived anthropogenic radionuclide contaminants. The Baltic Sea is one of the world’s largest brackish water basins with limited water exchange, making it an area particularly vulnerable to pollution. As a result of contamination by radioactive ^137^Cs after Chernobyl accident in 1986 and slow exchange of water between the Baltic Sea and the North Sea, it is the most polluted with ^137^Cs water body in the world (WOMARS 2005). Another important source of ^137^Cs in the Baltic Sea is the global fallout caused by nuclear weapon tests from the 1950s and 1960s (HELCOM 2009; Aarkrog 2003). The total input of ^137^Cs activity from Chernobyl to the Baltic Sea was estimated at 4 700 TBq while from the global fallout was 900 TBq (Ilus 2007). This isotope activity in the Baltic Sea is still higher than 30 years ago, before the failure at the Chernobyl nuclear power plant. Since 1985, continuous measurements of ^90^Sr and ^137^Cs are carried out to make sure that there is no future risk to humans and the environment. The Baltic Sea is one of the best-studied water bodies in terms of levels of ^137^Cs in various elements (water, sediment, fish, and other marine organisms) (HELCOM 1995, 2007, 2009; Ikaheimonen et al. 2009; Lujanienė et al. 2006; Saniewski and Zalewska 2016; Saremi et al. 2018; Zaborska et al. 2014; Zalewska 2012; Zalewska and Suplińska 2013a, b). Due to the need of the marine environmental contamination status assessment in terms of risk from the presence of polluting substances, including radioactive isotopes, it is critical to understand which organisms are the best bioindicators of specific radionuclides and would provide the most rapid warning of any potential leakage or movement into the environment. There are many papers on the application of organism as bioindicators. Nearly 25% of articles on natural bioindication deal with invertebrates, but only about 5% of them are related to radiation (Burger 2006). The representative of mussels *Mytilus edulis* is globally used as bioindicators for pollution with metals and radionuclides in the coastal environments. The reasons why *M. edulis* has been chosen is that it is globally distributed, it is commonly found in very dense populations in coastal waters, and it is also found in estuarine areas and in metropolitan harbours. There are currently very few studies dealing with isotopes levels in other benthic macroinvertebrates in Baltic Sea, which occurrence and abundance are influenced by changing chemical conditions. The oxygen and hydrogen sulphide content in the bottom layer of water is an important factor limiting macrozoobenthos occurrence (Rosenberg et al. 1992; Rosenberg 2001; Żmudziński and Osowiecki 1991). In the years 1956-1957, Mulicki and Żmudziński (1969), studied the decomposition of macrozoobenthos biomass and first discovered the presence of large surface “benthic deserts” (azoic areas) in the area of the Bornholm Deep and the Gdańsk Deep. Baltic benthic fauna is commonly exposed to waters of low oxygen concentration and toxic hydrogen sulphide (H_2_S), which in the sediments and even in the water column in the deeper parts of the Baltic Sea often occur. In the shallow bottom zone (0–25 m), wave activity, bottom and surface water currents and vertical mixing cause that the water above the sediments is well saturated with oxygen. At the deeper stations, below the halocline (50–70 m), the water temperature is lower than in the surface layer, and the salinity and water density are higher. Because the mixing of bottom water with well-oxygenated surface water is difficult, the only source of oxygen above the bottom are the inflows of oxygenated saline waters from the North Sea. However, as a result of organic-matter supply falling from the euphotic zone and affected on the top layer of the sediment and increase production of bacteria and consumption of oxygen and a decrease in the oxygen penetration depth and depleted over time (Feistel et al. 2008; Van Duyl et al. 1992). Deficiency of oxygen (hypoxia) or lack of it (anoxia) concerns mainly the regions of the southern Baltic deeps—Gdańsk, Bornholm and to a lesser extent the southern slope of the Gotland Deep. Nevertheless, even the most resistant species cannot survive prolonged periods of oxygen deficiency, which resulted in mass mortality of benthic fauna in many regions of the Baltic Sea and disappearance of all macrofauna from the region of the Gdańsk Deep (Diaz and Rosenberg 1995).

The main aim of the present investigation was to study the levels of ^137^Cs in the selected, most common benthic macroinvertebrates inhabiting southern Baltic Sea in the context of choosing the best bioindicators, taking into account their frequency and biomass. The results were used to calculate the dose rate from external and internal radiation, on the basis of which the potential hazard resulting from the presence of ^137^Cs in the Baltic Sea was assessed.

## Material and methods

### Study area and sampling

**Fig. 1:**
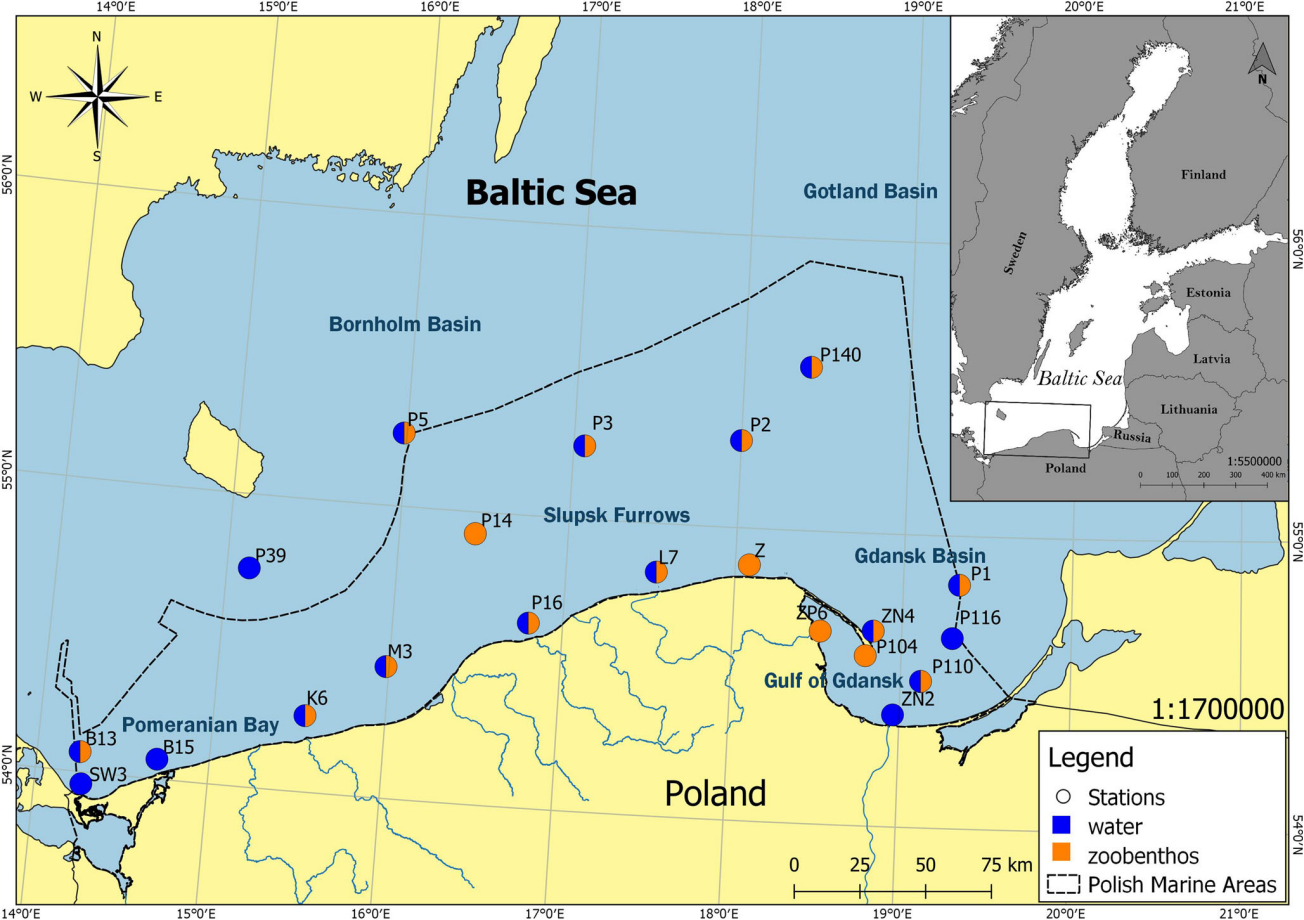
Location of sampling stations in the southern Baltic Sea

Benthic macroinvertebrates from the southern Baltic Sea were collected once a year at the beginning of June over 2011–2018 using van Veen grab sampler at 16 stations. Simultaneously seawater samples were collected once a year at the same time as macroinvertebrates at 2 m above the sea bottom with a rosette sampler, and accompanied by salinity and temperature profiling at 17 stations of which the complete parameters of water and macroinvertebrates were collected at 12 stations. Seawater samples of *ca.* 30 dm^3^ volume were acidified (200 ml 6M HCl) immediately after sampling and transported to the laboratory for further analysis. Besides, in 2018 surface sediment (0-10 cm) samples were collected at all macroinvertebrates stations for ^137^Cs activity analysis (Fig. 1).

### Laboratory analysis

^137^Cs activity in seawater was determined by gamma spectrometry. Twenty milligrams of Cs^+^ was added to each acidified seawater sample as a carrier. Caesium was absorbed in 10 g of ammonium phosphomolybdate (AMP) during the 20-min stirring, and the AMP was then separated by decantation and filtration, dried. The ^137^Cs activities were measured using a gamma spectrometry system. Extended Range Coaxial Ge Detectors (XtRa) model GX4018 (Canberra) with a relative efficiency of 40% and a resolution of 1.8 keV for the 1332 keV peak of ^60^Co were applied. The detector was coupled to an 8192-channel computer analyser and GENIE 2000 software. The measurements time for each sample was 80,000 s.

Macroinvertebrate and sediment samples were dried and homogenised. An integrated samples of macroinvertebrate (from all locations) of individual species for each year were measured, without divided into the size categories, which was connected with limited masses. Before analysis, the samples were ashed at a temperature of 450°C in a muffle furnace. After homogenisation, the ^137^Cs activity was measured using a gamma spectrometry as with water. The detector system was calibrated using the gamma mixed standards (Standard solution of gamma-emitting isotopes, code BW/Mix-γ/14/16). The radionuclides used in the reference solution during equipment calibration were ^241^Am, ^109^Cd, ^57^Co, ^51^Cr, ^113^Sn, ^85^Sr, ^137^Cs, ^54^Mn, ^65^Zn, and ^60^Co. This reference solution was used for preparing reference samples for equipment calibration. For equipment calibration, reference samples were analyzed in cylindrical plastic containers (40 mm diameter) with the same geometry as those used for environmental samples.

The reliability and accuracy of the measurements as well as comparability were verified by the participation in 2020 in the intercalibrations organized within the National Atomic Energy Agency in Poland (PAA) and analysis organized yearly by IAEA-MEL Monaco (Table 1).

**Table 1:** The reliability and accuracy of the measurements of laboratory

	Reported Laboratory Value [Bq dm^-3^]	Assigned value [Bq dm^-3^]	Accuracy	Precision	
^137^Cs	0.947 ± 0.009	0.908 ± 0.011	Pass	Pass	IAEA (S20NO50)
^137^Cs	3.00 ± 0.03	2.91 ± 0.15	Pass	Pass	PAA
	0.072 ± 0.03	0.071 ± 0.03	Pass	Pass	
	2.10 ± 0.18	1.77 ± 0.11	Pass	Pass	

### Assessment of exposure to radiation

The annual external dose rate to the respective marine organism were calculated using the site-specific activity concentrations according to Eqs. 1 and 2.

1 $$ {E}_{\mathit{\operatorname{ext}}(w)}={c}_w\ast {DCF}_{ext} $$

2 $$ {E}_{\mathit{\operatorname{ext}}(s)}={a}_s\ast {DCF}_{ext} $$

Herein are:

- *E*_*ext*(*w*)_ External dose rate from ^137^Cs in seawater to organism, in μGy h^-1^
- *E*_*ext*(*s*)_ External dose rate from ^137^Cs in the upper sediment layer to the organism,
- in μGy h^-1^
- *c*_*w*_ Activity concentration of radionuclide ^137^Cs measured in seawater, in Bq l^-1^
- *a*_*s*_ The specific activity of radionuclide ^137^Cs measured in the top layer of sediment, in Bq kg^-1^
- *DCF*_*ext*_ The dose conversion factor for external exposure of crustaceans (crab) (see ICRP 136, 2017) by radionuclide k, in μGy kg h^-1^ Bq^-1^

According to IAEA (2015), external exposure is influenced by the time organisms stay close to the seabed. This is reflected by Eq. 3.

3 $$ {E}_{ext}=\frac{x}{48\ \left[h\right]}\ast {E}_{\mathit{\operatorname{ext}}(s)}+\frac{48\ \left[h\right]-x}{48\ \left[h\right]}\ast {E}_{\mathit{\operatorname{ext}}(w)} $$

Herein are:

- *E*_*ext*_ External dose rate from radionuclide to species, in μGy h^-1^
- *x* The time during which the lower side of the organisms body receives external dose from ^137^Cs, in h

The same approach was used for the internal dose rates as shown in Eq. 4

4 $$ {E}_{int}={a}_f\ast {DCF}_{int} $$

Herein are:

- *E*_*int*_ The internal dose rate of ^137^Cs to species, in μGy h^-1^
- *a*_*f*_ The specific activity of ^137^Cs measured in species, in Bq kg^-1^
- *DCF*_*int*_ The dose conversion factor for internal exposure of crustaceans (crab) (see ICRP 136, 2017) by ^137^Cs, in μGy kg h^-1^ Bq^-1^

## Results

### Macroinvertebrates occurrence and biomass

**Fig. 2:**
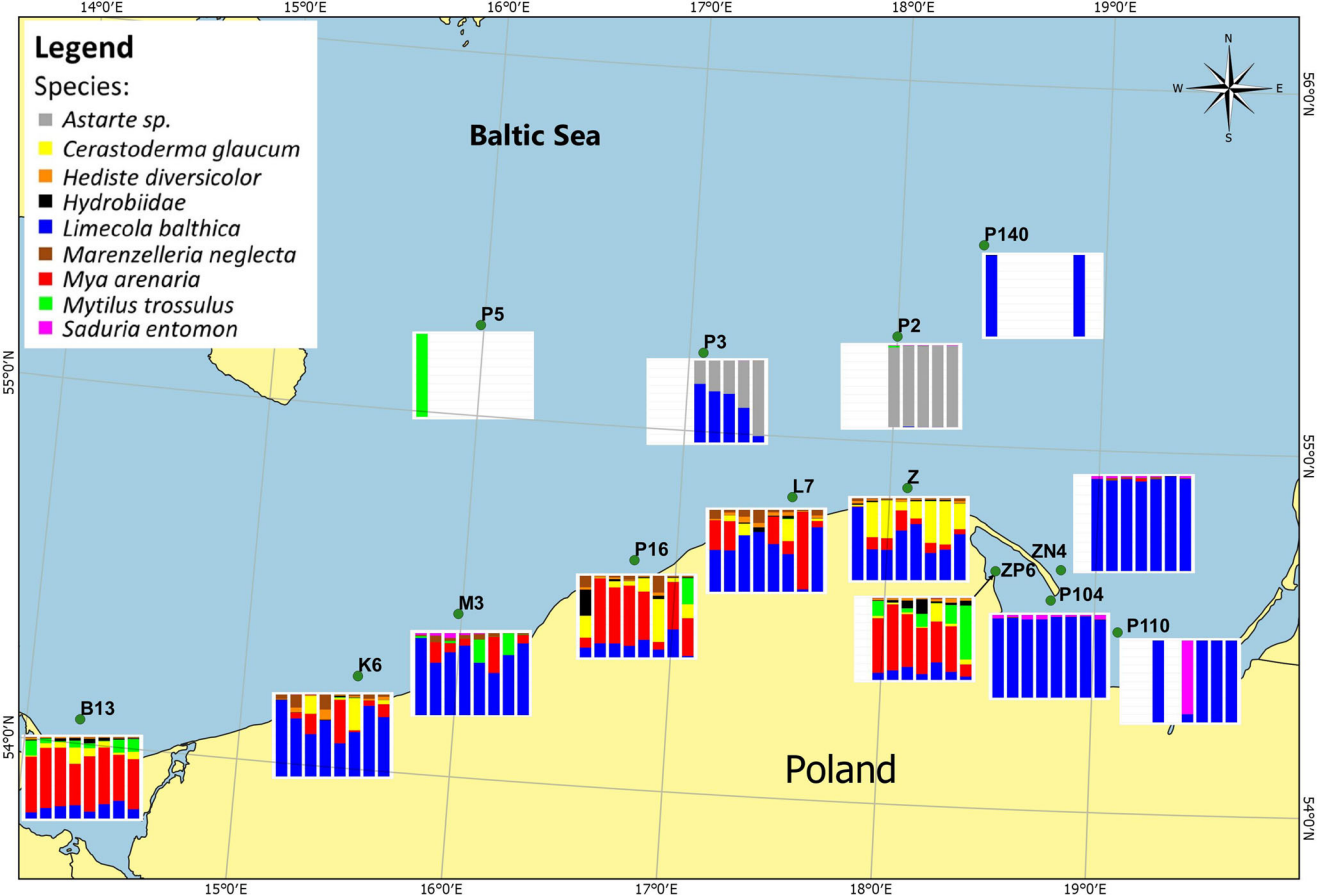
Percentage share in total biomass of macroinvertebrates at sampling stations in 2012-2018

The most frequently observed species in the southern Baltic Sea was *Limecola balthica*, which dominated in total biomass at stations K6, M3, L7, Z, P104, P110, ZN4, P140, while *Mya arenaria* had the largest share in total biomass at stations B13, P16, ZP6 and occasionally in years 2016 at station M3 and in 2017 at L7. At stations ZP6 and B13 *Mytillus trossulus* was dominant and accounting for 20% of total biomass (Fig. 2). *Astarte spp.* dominated in total biomass only at stations P2 and P3.

Average biomass of *L. balthica* ranged from 0.08 g_dw_.m^-2^ at station P140 to 103 g_dw_.m^-2^ at station P104. Each year at stations B13, K6, L7, M3, P110, P16, Z, ZN4, the observed biomass of *L. balthica* averaged to over a dozen g_dw_.m^-2^ only at P140, P2 stations the observed biomass was less than 1 g_dw_.m^-2^ (appendix). The biomass of *M. arenaria* ranged from 0.48 g_dw_.m^-2^ (ZN4 in 2014) to 286 g_dw_.m^-2^ (B13 in 2011). At stations B13, ZP6 and P16 total biomass of *M. arenaria* exceeded 10 g_dw_.m^-2^ in each year while at stations K6, L7, M3 and Z, the biomass of the species amounted to 1 g_dw_.m^-2^ and at station ZN4 it was close to 0. *M. trossulus* was observed at stations B13, ZP6,M3 (in 2017) and P16 (in 2018). At the remaining stations, its biomass was below 1 g. *Astarte spp.* was observed only at station P2, where its biomass ranged from 58.5 to 108 g_dw_.m^-2^ and station P3, where biomass of this species ranged from 1.71 to 13.0 g_dw_.m^-2^. *Cerastoderma glaucum* was most abundant at stations Z, B13 and P16 reaching average biomass levels in the period 2011–2018 ranging from 1.08 to 52.7 g_dw_.m^-2^ at station Z , from 1.45 to 22.82 g_dw_.m^-2^ at station B13 and from 0.87 to 21.9 g_dw_.m^-2^ at station P16 (appendix). At stations, K6 and L7 *C. glaucum* biomass was below 1 g_dw_.m^-2^. *Hydrobiideae* were observed occasionally at stations B13 and ZP6, reaching biomass ranging from 1 to 5 g_dw_.m^-2^ at other stations K6, L7, M3, P104, ZN4 average biomass in years 2011–2018 ranged from 0.004 g_dw_.m^-2^(M3) to 0.30 g_dw_.m^-2^ (L7). *Saduria entomon* occurred sporadically at stations P104, M3, ZN4, reaching biomass of 1–2 g_dw_.m^-2^, at stations K6, M3, P110, P2, P3, ZP6 biomass of this species was negligible. *Marenzelleria neglecta* was less pronounced in biomass, reaching 1 g_dw_.m^-2^ only at stations B13, L7 and Z, while at other stations, the species was observed only incidentally. *Hediste diversicolor* was observed in biomass ranging from 1 to 3 g_dw_.m^-2^ only at station ZP6 (appendix). At the station P1, throughout the research period, no macroinvertebrates organisms were present additionally. At P14 station with a rocky bottom, it was not possible to take samples using van Veen grab.

### ^137^Cs in bottom water

Average activity of ^137^Cs in bottom water in 2011–2018 calculated as the arithmetic mean for each year for 8 stations where macrozoobenthos occurred (ZN4, P3, M3, K6, P14, B13, P16, L7, P2, Z) changed from 32.1 ± 3.15 Bq m^-3^ in 2011 to 17.3 ± 1.68Bq m^-3^ in 2017. In 2018, a slight increase of ^137^Cs activity in bottom water was observed compared to the previous year (18.5 ± 2.70 Bq m^-3^) (Table 2). Throughout the period, the lowest activity was observed in the bottom waters of the Bornholm Basin (P5), being heavily influenced by inflows from the North Sea, which play a significant role in determining environmental conditions.

**Table 2:** The activity of ^137^Cs in bottom water in 2011–2018

	depth [m]	2011	2012	2013	2014	2015	2016	2017	2018
^137^Cs [Bq m^-3^]									
P3	87	26.6	24.1	20.3	17.3	17.8	18.3	14.9	18.4
P5	87	25.4	18.9	24.2	18.7	14.3	17.8	12.4	15.2
P39	61	28.9	27.3	23.0	30.2	16.7	18.8	16.0	18.8
P16	19	29.9	32.4	22.4	24.7	20.2	22.1	17.8	17.0
M3	36	32.5	36.9	28.8	32.0	20.2	23.6	17.8	18.4
K6	13	33.4	30.5	24.3	33.9	21.3	21.9	18.5	16.9
B13	12	33.7	27.8	22.4	26.0	18.8	20.8	16.9	19.3
B15	13	33.9	27.3	25.8	24.6	15.2	24.2	17.5	18.5
SW3	10	32.5	24.6	22.0	22.4	17.6	19.1	15.7	16.9
P140	86	30.4	32.3	26.6	34.9	19.6	20.0	17.2	19.4
P2	74	28.9	28.0	26.3	23.7	17.9	23.3	15.0	25.2
Ł7	21	34.4	33.0	26.9	23.5	17.8	22.1	20.3	17.0
P1	105	26.4	33.9	26.7	23.0	18.5	23.2	17.1	27.8
P110	66	29.5	35.2	26.2	27.3	21.2	21.40	17.7	17.0
P116	86	30.1	34.9	29.7	35.3	19.1	21.3	17.8	20.2
ZN4	68	37.1	35.6	29.7	31.4	19.2	23.3	17.5	16.1
ZN2	15	31.8	35.4	23.5	22.8	19.5	21.8	18.3	21.6
Average activity in Southern Baltic Sea		30.9 ± 3.07	30.5 ± 4.86	25.2 ± 2.69	26.6 ± 5.33	18.5 ± 1.85	21.3 ± 1.90	17.0 ± 1.72	19.0 ± 3.15
Average activity on stations where macrozoobenthos occurred		32.1 ± 3.15	31.0 ± 4.02	25.1 ± 3.12	26.6 ± 5.19	19.2 ± 1.23	21.9 ± 1.62	17.3 ± 1.68	18.5 ± 2.70

### ^137^Cs in bottom sediment

In 2018, the activity of ^137^Cs was at a lower level at 11 stations dominated by sand or clay sediment, where macrozoobenthos organisms are the most numerous (ZN4, P3, M3, K6, P14, B13, P16, L7, P2, Z, P104). They range from 1.1 Bq kg^-1^ dw at station L7 and K6 to 14.7 Bq kg^-1^ dw at station P104 (Table 3). At four stations in the deep water region (P5, P110, P1, P140), where the biomass of organisms was negligible, the activities of ^137^Cs in muddy sediments were highest and ranged from 47.6 Bq kg^-1^ dw at station P5 to 170 Bq kg^-1^ dw at station P140.

**Table 3:** The activity of ^137^Cs in sediment in 2018

	ZN4			P3			M3			K6			P14		
granulometry	Activity [Bq kg^-1^]		LOI	Activity [Bq kg^-1^]		LOI	Activity [Bq kg^-1^]		LOI	Activity [Bq kg^-1^]		LOI	Activity [Bq kg^-1^]		LOI
	in sieve size	average		in sieve size	average		in sieve size	average		in sieve size	average		in sieve size	average	
0.5	24.7 ± 1.64	10.5	6.25	8.55 ± 0.91	9.80	1.19	15.1 ± 1.47	3.27	0.21	2.94 ± 0.56	1.10	0.2		1.89	
0.25	10.3 ± 1.36		6.47	7.68 ± 0.75		1.21	4.86 ± 0.71		2.49	0.58 ± 0.47		0.14	<0.83		0.17
0.125	10.7 ± 0.80		0.93	8.36 ± 0.83		1.72	2.41 ± 0.63		0.65	<0.85		0.14	2.13 ± 0.56		0.20
0.0625	7.08 ± 0.66		0.90	24.0 ± 1.36		3.95	5.02 ± 1.28		0.62				<1.30		0.42
<0.0625	22.6 ± 1.26		3.79	34.2 ± 1.79		6.88									
	B13			P16			L7			P2			Z		
granulometry	Activity [Bq kg^-1^]		LOI	Activity [Bq kg^-1^]		LOI	Activity [Bq kg^-1^]		LOI	Activity [Bq kg^-1^]		LOI	Activity [Bq kg^-1^]		LOI
	in sieve size	average		in sieve size	average		in sieve size	average		in sieve size	average		in sieve size	average	
0.5		1.29			0.81		2.60 ± 0.64	1.06	0.21	7.47 ± 0.81	8.86	1.39	0.80 ± 0.51	1.73	0.25
0.25	1.42 ± 0.52		0.38	0.57 ± 0.40		0.13	0.87 ± 0.51		0.16	6.27 ± 0.76		1.1	1.90 ± 0.56		0.26
0.125	1.19 ± 0.51		0.42	1.08 ± 0.58		0.29	1.07 ± 0.56		0.16	8.73 ± 0.80		1.27	1.51 ± 0.64		0.41
0.0625	2.59 ± 0.54		0.61	1.60 ± 0.63		0.46	1.68 ± 1.30		0.21	13.3 ± 0.89		1.99	3.39 ± 0.74		0.59
<0.0625										43.4 ± 2.22		5.16			
	P104			P5			P110			P1			P140		
granulometry	Activity [Bq kg^-1^]		LOI	Activity [Bq kg^-1^]		LOI	Activity [Bq kg^-1^]		LOI	Activity [Bq kg^-1^]		LOI	Activity [Bq kg^-1^]		LOI
	in sieve size	average		in sieve size	average		in sieve size	average		in sieve size	average		in sieve size	average	
0.5		14.7		47.8 ± 1.75	47.6	13.83		170		5.05 ± 1.94	69.9	14.9		114	
0.25	7.21 ± 0.74		0.89	47.2 ± 1.62		13.97	168 ± 3.56		16.6	60.6 ± 2.17		15.6	116 ± 2.64		11.9
0.125	14.9 ± 1.75		0.65	44.3 ± 1.78		14	188 ± 3.93		17.5	77.1 ± 2.68		16.6	128 ± 2.94		12.3
0.0625	24.2 ± 1.24		3.78	46.8 ± 1.94		12.83	180 ± 3.84		17.8	95.2 ± 3.71		17.7	115 ± 2.963		12.8
<0.0625	43.1 ± 1.67		20.11	52.1 ± 2.17		14.13	160 ± 3.64		14.4	82.3 ± 2.86		17.1	103 ± 2.91		10.5

### ^137^Cs in macroinvertebrates

Taking into account 35 macroinvertebrates species found in the southern Baltic Sea, only in the case of 9 species, the biomass enabled isotope analysis. The average dry weight integrated species from each year used for the analysis was *M. trossulus* 11.3 g (from 3.95 to 16.6 g)*, M. neglecta* 2.44 g (from 0.87 to 4.46 g)*, Astarte* sp*.* 40.8 g (from 30.1 to 49.3 g)*, C. glaucum* 18.6 g (from 7.24 to 31.7 g)*, L. balthica* 43.8 g (from 33.9 to 59.1 g)*, M. arenaria* 44.4 g (from 27.6 to 58.3 g)*, Hydrobiidae* 2.18 g (from 0.81 to 3.37 g)*, H. diversicolor* 2.73 g (from 0.58 to 7.91 g)*, S. entomon* 2.01 g (from 0.22 to 4.31 g) (Table 4). The lowest ^137^Cs concentrations were characteristic for *M. trossulus*, and they stay in the range from 0.37 to 0.60 Bq kg^-1^ dw. The widest range of activities was observed *in M. neglecta* from 3.79 to 42.5 Bq kg^-1^ dw. Shell organisms *Astarte* sp*.* (2.19 Bq kg^-1^ dw), *C. glaucum* (1.48 Bq kg^-1^ dw), *L balthica* (1.86 Bq kg^-1^ dw), *M. arenaria* (1.33 Bq kg^-1^ dw), and *M. trossulus* (0.52 Bq kg^-1^ dw) were characterised by the lowest average activity of ^137^Cs. Definitely higher average activities were observed in the case of organisms without shell, like *H. diversicolor* (5.11 Bq kg^-1^ dw), *M. neglecta* (19.9 Bq kg^-1^ dw) and *S. entomon* (11.9 Bq kg^-1^ dw) (Tab. 4).

**Table 4:** The activity concentration of ^137^Cs in macroinvertebrates ± measurement error in 2011–2018

	2011	2012	2013	2014	2015	2016	2017	2018
	^137^Cs [Bq kg^-1^dw.]							
*Astarte sp*.				3.53 ± 0.17	3.29 ± 0.18	1.52 ± 0.28	1.82 ± 0.27	0.81 ± 0.21
d.w of sample [g]				49.3	48.1	30.1	37.7	38.6
*Cerastoderma glaucum*				3.41 ± 0.53	2.40 ± 0.30	0.86 ± 0.21	0.54 ± 0.17	0.22 ± 0.17
d.w of sample [g]				26.0	13.8	31.7	7.24	14.1
*Hediste diversicolor*		3.89 ± 1.43	9.34 ± 1.98	5.36 ± 2.00		7.31 ± 2.61	2.3 ± 1.12	2.51 ± 1.08
d.w of sample [g]		3.04	2.12	0.58		1.82	0.94	7.91
*Hydrobiidae*		18.7 ± 3.39	14.4 ± 2.59	14.0 ± 4.77		10.2 ± 2.58	8.03 ± 5.09	2.64 ± 1.25
d.w of sample [g]		1.20	2.78	3.37		2.47	0.81	2.45
*Limecola balthica*	2.22 ± 0.17	2.06 ± 0.17	2.02 ± 0.18	1.7 ± 0.17	1.67 ± 0.21	2.16 ± 0.21	1.11 ± 0.26	1.47 ± 0.26
d.w of sample [g]	59.1	58.1	41.0	44.3	43.1	33.9	34.4	36.6
*Marenzelleria neglecta*		28.5 ± 1.00	42.5 ± 5.89	25.7 ± 10.6	26.6 ± 10.8	6.78 ± 2.52	5.41 ± 1.58	3.79 ± 1.27
d.w of sample [g]		4.46	1.56	1.61	2.61	3.41	0.87	2.54
*Mya arenaria*	1.10 ± 0.16	0.58 ± 0.12	1.56 ± 0.26	3.29 ± 0.17	2.24 ± 0.14	1.14 ± 0.14	0.39 ± 0.27	0.34 ± 0.20
d.w of sample [g]	56.5	55.6	58.3	36.2	41.4	33.6	27.6	45.8
*Mytilus trossulus*	0.46 ± 0.18	0.6 ± 0.21		0.58 ± 0.17			0.37 ± 0.22	0.57 ± 0.22
d.w of sample [g]	16.6	14.9		3.95			8.57	12.5
*Saduria entomon*		14.8 ± 3.40	15.7 ± 0.94	12.7 ± 1.15	19.5 ± 6.93	6.99 ± 3.35	1.62 ± 0.48	
d.w of sample [g]		0.22	4.16	2.52	1.21	0.47	1.19	

### Concentration ratio (CR)

To determine the bioindication potential of organisms, there were calculated concentration ratio (CR) based on the activities of ^137^Cs in organisms (fresh weight) every year divided by weighted average ^137^Cs activity (Bq dm^-3^) in bottom seawater corresponding to the years of sampling macroinvertebrates.

5 $$ CR=\frac{\mathrm{Cm}}{\mathrm{Cw}} $$

where:

- CR – bioconcentrations factors (dm^3^ kg^-1^_fw_),
- Cm -^137^Cs activity (Bq kg^-1^_fw_) in macroinvertebrate
- Cw -^137^Cs activity (Bq dm^-3^) in bottom seawater

The average values of CR in macroinvertebrate in 2011–2018 has changed in terms *M. trossulus* (9.09 ± 2.23 dm^3^ kg^-1^_fw_) < *M. arenaria* (25.7 ± 17.3 dm^3^ kg^-1^_fw_) < *H. diversicolor* (29.4 ± 14.4 dm^3^ kg^-1^_fw_) < *L. balthica* (42.9 ± 8.89 dm^3^ kg^-1^_fw_) < *C. glaucum* (42.9 ± 32.3 dm^3^ kg^-1^_fw_) < *Astarte sp*. (67.9 ± 30 dm^3^ kg^-1^_fw_) < *M. neglecta* (127 ± 90.7 dm^3^ kg^-1^_fw_) < *Hydrobiidae* (237 ± 80.8 dm^3^ kg^-1^_fw_) < *S. entomon* (314 ± 191 dm^3^ kg^-1^_fw_) (Table 5). The lowest variability in the years were found for bivalves: *L*. *balthica* from 31.6 to 56.9 dm^3^ kg^-1^_fw_ and in *M*. *trossulus* from 5.86 to 12.33 dm^3^ kg^-1^_fw_. The biggest fluctuation of in value of CR in the period 2011–2018 characterized *M. neglecta from* 13.8 to 138 dm^3^ kg^-1^_fw_ and *C. glaucum* from 7.47 to 86.2 dm^3^ kg^-1^_fw_ .

**Table 5:** Concentration ratio of ^137^Cs in macroinvertebrates in 2011–2018

	2011	2012	2013	2014	2015	2016	2017	2018	arithmetical mean ± standard division
	CR [dm^3^ kg^-1^_fw_]								
*Astarte sp*.				78.2	119	43.8	66.3	32.6	67.9 ± 30.0
*Cerastoderma glaucum*				86.2	77.3	24.1	19.6	7.47	42.9 ± 32.3
*Hediste diversicolor*		14.9	49.8	28.3		47.6	15.0	21.1	29.4 ± 14.4
*Hydrobiidae*		308	291	295		234	222	70.7	237 ± 80.8
*Limecola balthica*	34.8	31.6	43.8	35.3	51.7	56.9	37.5	51.5	42.9 ± 8.89
*Marenzelleria neglecta*		138	276	153	217	54.6	35.8	13.8	127 ± 90.7
*Mya arenaria*	16.2	8.82	35.6	57.2	46.3	21.4	9.79	10.2	25.7 ± 17.3
*Mytilus trossulus*	5.86	7.49		10.2			9.57	12.3	9.09 ± 2.23
*Saduria entomon*		314	382	251	679	197	63.1		314 ± 191

### Total doses to macroinvertebrates

**Fig. 3:**
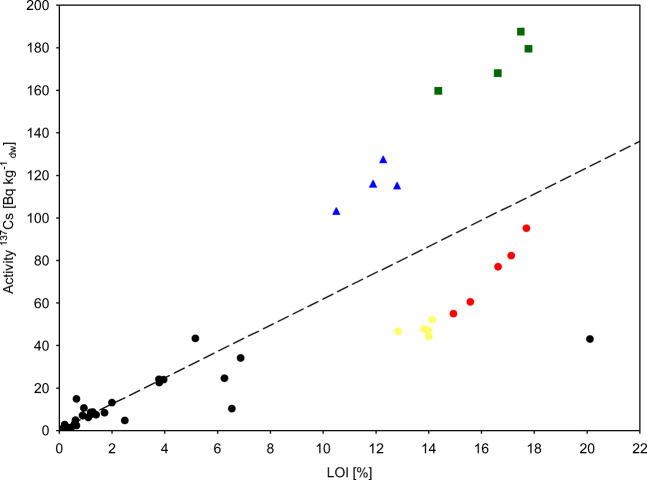
Correlation of 137Cs activity in relation to LOI in sediments (P1 - red circle, P5 - yellow circle, P110 - green square, P140 - blue triangle)

Based on the data on the concentrations of ^137^Cs in individual elements, an attempt was made to estimate the dose rates to which species is exposed from external and internal sources. For calculate the dose rates, the values of isotopes concentrations in water, sediment and species were used. Calculations were carried out upon 2018 data. The activity of ^137^Cs in an integrated sample of the species from different stations was used for the calculations. In the case of bottom water and sediments, the weighted average activity of ^137^Cs from stations where each species were located, taking into account the percentage of individuals from each station in the total weight of the sample used for analyzes. Using the formulas 1–4 the total doses from external and internal sources to macroinvertebrates in 2018 were 1.35 nGy h^-1^ in the case of *Astarte sp.*, 0.21 nGy h^-1^*C. glaucum*, 0.66 nGy h^-1^*H. diversicolor*, 0.68 nGy h^-1^*Hydrobiidae*, 3.29 nGy h^-1^*L. balthica*, 0.97 nGy h^-1^*M. neglecta*, 0.23 nGy h^-1^*M. arenaria*, 0.28 nGy h^-1^*M. trossulus*, 2.03 nGy h^-1^*S. entomon*(Table 6). In the case of organisms living mainly on sandy bottoms (*M. arenaria*, *C. glaucum*, *Saduria entemon*), the internal dose accounted for approximately 15-30% of the total dose. For species *H. diversicolor*, *M. neglecta*, *Hydrobiidae* internal dose accounted for approximately 70% of the total dose. In the case of *L. balthica* external dose almost constituted 92% of the total dose, which was caused by occurence of these organisms both on the sandy bottom and on the accumulation bottom (P110) where that activity of ^137^Cs is higher due to the higher share of organic matter (Fig. 3, Table 3).

**Table 6:** Total dose to macroinvertebrates from ^137^Cs in 2018

	E_int(j,k)_	E_ext(j,k)_	Dose
	nGy h-1		
*Astarte sp.*	0.15	1.20	1.35
*Cerastoderma glaucum*	0.04	0.00	0.04
*Hediste diversicolor*	0.45	0.21	0.66
*Hydrobiidae*	0.48	0.21	0.68
*Limecola balthica*	0.26	3.02	3.29
*Marenzelleria neglecta*	0.68	0.29	0.97
*Mya arenaria*	0.06	0.17	0.23
*Mytilus trossulus*	0.10	0.18	0.28
*Saduria entomon*	0.29	1.74	2.03

## Discussion

^137^Cs in benthic invertebrates comes from three major sources of contamination (seawater, food, and sediments) (Metian et al. 2011; Topcuoğlu 2001). The size of the sediment fraction did not affect the activity of ^137^Cs in the sediment (Table 3). The main factor responsible for the activity was the share of organic matter (*r* = 0,82, *p*<0.05) (Fig. 3). Therefore, in the case of the bottom areas with intensive sedimentation processes associated with the transportation type of bottom (LOI—loss on ignition, values of 4–10%) and the accumulation bottom (with LOI values >10%) (Håkanson et al. 2003), they are characterized by large proportion of organic matter which would possibly affect ^137^Cs mobility (Nakamaru et al. 2007). At stations (P110, P1, P140) located in Gulf of Gdańsk which are influenced by the Vistula river, which is second largest river, after Neva, draining into the Baltic Sea, and sedimentation processes in this area it may even be 500g m^-2^ year^-1^ are characterized by high activity of ^137^Cs (Zalewska et al. 2020). Therefore, in the shallow water station where macrozoobenthos are abundant, the sediments are characterized by a low activity of ^137^Cs in the depths part of the Baltic Sea only small numbers of tolerant species of the *Bylgides sarsii* was noted. The highest diversities, including species *C. glaucum H. diversicolor*, *Hydrobiidae*, *M. neglecta and M. arenaria* are characteristic of the sandy bottom down to a depth of 20 m in the Pomeranian Bay and down to 25 m in the open sea (Fig. 2, appendix). *S. entomon, Astarte* sp. communities are reaching to the depth of the halocline 50–60 m in the Bornholm Basin and the western part of Słupsk Furrow, 70 m in the eastern part of Słupsk Furrow, 80 m in the Gdańsk Basin and Gotland Basin. *L. balthica* and *M. trossulus* was found both in the shallow coastal waters and deep-sea areas of eastern Gotland Basin and Gdańsk Basin.

**Fig. 4:**
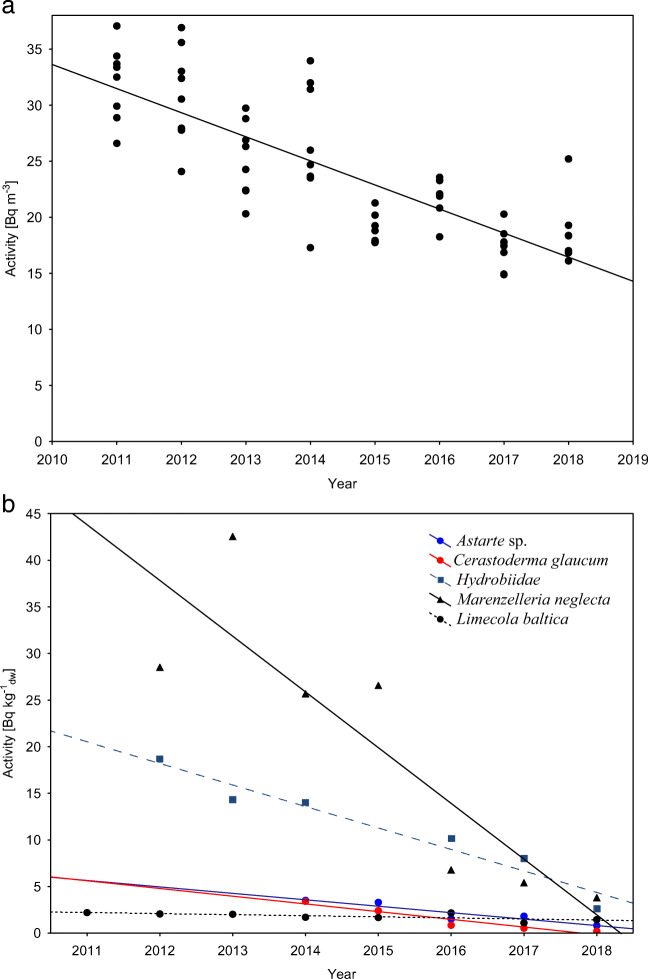
The activity of 137Cs in the southern Baltic Sea between 2011 - 2018; **a**) near-bottom water, **b**) macroinvertebrates

After Chernobyl accident ^137^Cs concentrations in seawater increased until 1991, when the average value in the southern Baltic Sea was of the order of 101 Bq m^− 3^, which was caused by the inflow of more polluted waters from the northern Baltic as well as to considerable riverine discharges. Since 1991, ^137^Cs activity has been falling as a consequence of radioactive decay, the inflow of less polluted waters from the North Sea, the inflow of freshwater from the Baltic Sea catchment area, sorption onto suspended particulate matter, deposition in sediments and bioaccumulation (Zalewska and Suplińska 2013a). The cumulative effects of all those factors account for the effective half-lives of ^137^Cs in seawater to be 9 years, despite the fact that their real half-life is close to 30 (Saniewski and Zalewska 2018) In the period 1991–2010, a statistically significant decrease in the activity of ^137^Cs in water was observed (*r* = 0.94, *p*<0.05). The average activity in southern Baltic waters decreased from 101 Bq m^-3^ in 1991 to 35.8 Bq m^-3^ in 2010. In the period 2011–2018, a statistically significant decreased trend was continued (*r*= -0.80 *p*<0. 05) and the average activity decreased from 31.3 Bq m^-3^ in 2011 to 19.8 Bq m^-3^ in 2018 (Fig. 4a). As a consequence of changes in water column, decreased trend of the activity ^137^Cs in sediment was also observed, and therefore both processes cause a decrease of activity in organisms. A similar downward trend in ^137^Cs activity was observed with time in all studied organisms, but only in five: *Hydrobiidae* (*r* = -0,97, *p*<0. 05), *C. glaucum* (r = -0,96, *p*<0. 05), *Astarte* sp. (*r*= -0,936, *p*<0. 05), *M. neglecta* (*r* = -0,88, *p*<0. 05), *L. baltica* (*r* = -0.73, *p*<0. 05) the trend was statistically significant (Fig. 4b). Nevertheless, there are no clear decrease trends in the case of *Mytilus* which is globally used as bioindicators (Table 4). In our research, the size of the organisms was not included in the analyses, but it could have an impact on the activity. In the years 2004–2018, the average shell size of *M*. *trossulus* collected in the Gulf of Gdańsk varied from 38 to 44 mm. In the case of individuals from the coastal waters of the Bornholm Basin in the vicinity of the Rowy port, the average size of the mussels was much smaller and ranged from 17 to 30 mm, and at the same time by 2014 there was a decrease in the average size of organisms compared to the previous year. Similarly at stations where *M*. *neglecta* dominates, an increase in the average number of individuals was observed at station P16 from 200 individuals m^-2^ in 2011 to 695 individuals m^-2^ in 2018, at station Z from 415 individuals m^-2^ in 2011 to 646 individuals m^-2^ in 2018 and L7 from 410 individuals m^-2^ in 2011 to 497 individuals m^-2^ in 2018 with no increase in the total biomass. At all three stations, a downward trend was observed in the average weight of single species P16 (*r* = -0.53), Z (*r* = -0.59) and L7 (*r* = -0.29). Therefore, a significant decrease in the measured activity of ^137^Cs in samples from 2016-2018 could have been caused by a greater number of smaller *M*. *neglecta* specimens, what can be directly connected with the age of specimens and shorter bioaccumulation time. Growth and metabolism depend on age so specific phenomenon called the “size effect” allow extends exposure to hazardous substances which are taken up from water (Catsiki and Florou 2006; Kryshev and Ryabov 2000). For all organisms the correlation of average ^137^Cs activities with those in bottom seawater was observed *M. trossulus* (*r* =0.42)*, M. neglecta* (*r* =0.61), *Astarte* sp*.* (*r* =0.42), *C. glaucum* (*r* =0.69), *M. arenaria* (*r* =0.17)*, H. diversicolor* (*r* =0.36), *S. entomon* (*r* =0.40), but only in *L. baltica* (*r*=0.75, *p*<0.05) and *Hydrobiidae* (*r* =0.88, *p*<0.05) correlation was statistically significant.

**Fig. 5:**
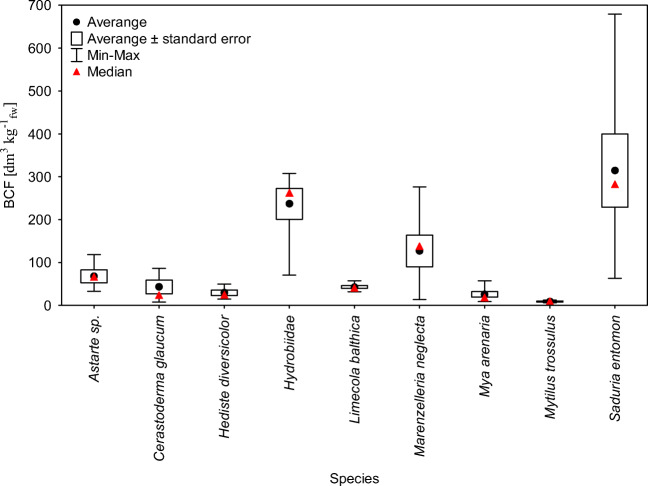
Concentration ratio (CR) for investigated organisms

Taking into account a narrow range of CR values, mainly *L. balthica*, *M. arenaria*, and *M. trossulus* could be considered as potential bioindicators (Fig. 5). By comparing the average CR values for *M. trossulus* (9.09 dm^3^ kg^-1^_fw_) and *L. balthica* (42.9 dm^3^ kg^-1^_fw_) based on data from 2011 to 2018 with literature values calculated for the same species in 2004 were (110 dm^3^ kg^-1^_dw_, 68 dm^3^ kg^-1^_dw_ respectively) (Zalewska and Saniewski 2011; Zalewska and Suplińska 2013a) and taking into account the share of dry matter in those species 42% and 58% respectively, only in the case of *L. balthica* CR values are similar. Differences in the CR values for *M. trossulus* could be caused by the size of individuals, which could effect on the accumulation of ^137^Cs. In the case of organisms *M. neglecta*, *Hydrobiidae*, *H. diversicolor*, and *S. entomon*, the CR values were much higher and stayed in the larger range, what was most likely related to the way of feeding, as well as a change in the size of individuals. *C. glaucum*, *L. balthica*, *M. arenaria* feed mostly particulate organic matter (POM), microalgae that filters out of volumes of water, based on concentration δ^15^N used to calculate the tropic level their trophic level is 2,09–2,32. *M. neglecta*, *H. diversicolor*, and *Hydrobiidae* feed sediment organic matter (SOM), microalgae, live and dead, animal tissue and plants. Values of stable isotopes ratios of nitrogen for these organisms are higher and the trophic level of these species calculated on the basis of δ15N is 2,56–2,71 (Jędruch et al. 2019). It makes the enrichment in ^137^Cs in relation to water is higher than in typical filterers as a result bioaccumulation and enrichment on trophic transfer (Helda et al. 2003; Thomas et al. 2018).

For all species the total doses (Table 6) were lower than the lower range of the derived consideration reference levels (DCRL), which is equal to 40 μGy h^-1^(ICRP 2008). This means that it can be assumed that the impact of radiation is negligible.

## Conclusions

Taking into account the narrow range of CR coefficients, statistically significant correlation between ^137^Cs activities in seawater and species, a high frequency of occurrence and thus high biomass and the common occurrence, only *L. balthica* could potentially serve as indicators for shallow and deep-sea areas accordingly. Widely spread *M. trossulus* was characterised by large size variability in samples which may impact on bioaccumulation and shows no significant correlation between activities in species and seawater and appears not so common as *L. balthica*.

## Supplementary information

ESM 1 (DOCX 34 kb)

## Data Availability

Not applicable.

## Declarations

### Conset for publication

Not applicable. This manuscript does not contains any individual person’s data in any form.

## References

[CR1] Aarkrog A (2003). Input of anthropogenic radionuclides into the world ocean. Deep-Sea Res Part II Top Stud Oceanogr.

[CR2] Burger J (2006). Bioindicators: A Review of Their Use in the Environmental Literature 1970–2005. Environ Bioindic.

[CR3] Catsiki V-A, Florou H (2006). Study on the behavior of the heavy metals Cu, Cr, Ni, Zn, Fe, Mn and 137Cs in an estuarine ecosystem using Mytilus galloprovincialis as a bioindicator species: the case of Thermaikos gulf, Greece. J Environ Radioact.

[CR4] Diaz RJ, Rosenberg R (1995). Marine benthic hypoxia: review of its ecologicaleffects and the behavioural responses of benthic macrofauna. Oceanogr Mar Biol Annu Rev.

[CR5] Feistel R, Feistel S, Nausch G, Szaron J, Łysiak-Pastuszak E, Ærtebjerg G (2008) Baltic: Monthly time series 1900 – 2005. [in:] Feistel R., Nausch G., Wasmund N. (eds.), 2008. State and evolution of the Baltic Sea, 1952-2005. Wiley Interscience – A John Wiley & Sons, Inc Publication: 311-336.

[CR6] Håkanson L, Lundin L.-C., Savchuk O, Ionov V, Musielak S, Furmanczyk K (2003) The Baltic Sea. W: Environmental Science (red. L. Ryden, P. Migula, M. Andersson), 120–147. The Baltic University Press, Uppsala

[CR7] HELCOM (1995) Radioactivity in the Baltic Sea 1984–1991, Baltic Sea Environ. Proc. No. 61, p. 182

[CR8] HELCOM (2007) Long-lived radionuclides in the seabed of the Baltic Sea. Report of the Sediment Baseline Study of HELCOM MORS-PRO in 2000–2005. Baltic Sea Environment Proceedings No. 110.

[CR9] HELCOM (2009) Radioactivity in the Baltic Sea 1999-2006. Baltic Sea Environment Proceedings No.117,

[CR10] Heldal HE, Føyn L, Varskog P (2003). Bioaccumulation of 137Cs in pelagic food webs in the Norwegian and Barents Seas. J Environ Radioact.

[CR11] IAEA (2015) International Atomic Energy Agency - TECDOC 1759: Determining the suitability of materials for disposal at sea under the London Convention 1972 and London Protocol 1996: a radiological assessment procedure. — Vienna: International Atomic Energy Agency, 2015. 100 pp., ISBN 978–92–0–100215–0

[CR12] ICRP, 2008. International Commission on Radiological Protection – Publication 108: Environmental Protection: the Concept and Use of Reference Animals and Plants. Annals of the ICRP 108/4-6

[CR13] Ikaheimonen TK, Outola I, Vartti VP, Kotilainen P (2009). Radioactivity in the Baltic Sea: inventories and temporal trends of 137Cs and 90Sr in water and sediments. J Radioanal Nucl Chem.

[CR14] Ilus E (2007). The Chernobyl accident and the Baltic Sea. Boreal Environ Res.

[CR15] Jędruch A, Bełdowska M, Ziółkowska M (2019). The role of benthic macrofauna in the trophic transfer of mercury in a low-diversity temperate coastal ecosystem (Puck Lagoon, southern Baltic Sea). Environ Monit Assess.

[CR16] Kryshev A, Ryabov I (2000). A Dynamic model of accumulation by fish of different age classes. J Environ Radioact.

[CR17] Lujanienė, G., Jokšas, K., Šilobritienė, B., & Morkūnienė, R. 2006. Physical and chemical characteristics of 137Cs in the Baltic Sea. Radioact Environ, 165–179. 10.1016/s1569-4860(05)0801

[CR18] Metian M, Warnau M, Teyssié JL, Bustamante P (2011). Characterisation of 241Am and 134Cs bioaccumulation in the King scallop Pecten maximus: investigation via three exposure pathways. J Environ Radioact.

[CR19] Mulicki Z, Żmudziński L (1969) Resources of the South Baltic zoobenthos (in the years 1956-1957). Works of National Marine Fisheries Research Institute. Gdynia, 77-101 (in Polish).

[CR20] Nakamaru Y, Ishikawa N, Tagami K, Uchida S (2007). Role of soil organic matter in the mobility of radiocesium in agricultural soils common in Japan. Colloids Surf. A Physicochem Eng Aspects.

[CR21] Rosenberg R, Loo L-O, Möller P (1992). Hypoxia, salinity and temperature as structuring factors for marine benthic communities in a eutrophic area. Neth J Sea Res.

[CR22] Rosenberg R (2001). Marine benthic faunal successional stages and related sedimentary activity. Sci Mar.

[CR23] Saniewski M, Zalewska T (2016). Atmospheric deposition and riverine load of ^90^Sr and ^137^Cs to the Gulf of Gdańsk (southern Baltic Sea) in the period 2005-2011. J Environ Radioact.

[CR24] Saniewski M, Zalewska T (2018). Budget of 90 Sr in the Gulf of Gdańsk (southern Baltic Sea). Oceanologia.

[CR25] Saremi S, Isaksson M, Harding KC (2018). Bio accumulation of radioactive caesium in marine mammals in the Baltic Sea – Reconstruction of a historical time series. Sci Total Environ.

[CR26] Topcuoğlu S (2001). Bioaccumulation of cesium-137 by biota in different aquatic environments. Chemosphere.

[CR27] Thomas DM, Lee C-S, Fisher NS (2018). Bioaccumulation and trophic transfer of 137 Cs in marine and freshwater plankton. Chemosphere..

[CR28] WOMARS (2005) Worldwide marine radioactivity studies Radionuclide levels in oceans and seas. International Atomic Energy Agency IAEA-TECDOC-1429, Vienna.

[CR29] Van Duyl FC, Kop AJ, Kok A, Sandee AJJ (1992). The impact of organic matter and macrozoobenthos on bacterial and oxygen variables in marine sediment boxcosms. Neth J Sea Res.

[CR30] Zaborska A, Winogradow A, Pempkowiak J (2014). Caesium-137 distribution, inventories and accumulation history in the Baltic Sea sediments. J Environ Radioact.

[CR31] Zalewska T, Saniewski M (2011). Bioaccumulation of 137Cs by benthic plants and macroinvertebrates. Oceanol Hydrobiol Stud.

[CR32] Zalewska T (2012). Seasonal changes of 137Cs in benthic plants from the southern Baltic Sea. J Radioanal Nucl Chem.

[CR33] Zalewska T, Suplińska M (2013). Anthropogenic radionuclides 137Cs and 90Sr in the southern Baltic Sea ecosystem. Oceanologia.

[CR34] Zalewska T, Suplińska M (2013). Fish pollution with anthropogenic 137Cs in the southern Baltic Sea. Chemosphere.

[CR35] Zalewska T, Przygrodzki P, Suplińska M, Saniewski M (2020). Geochronology of the southern Baltic Sea sediments derived from 210Pb dating. Quat Geochronol.

[CR36] Żmudziński L, Osowiecki A (1991). Long-term changes in macrozoobenthos of the Gdańsk Deep. Int Rev Gesamten Hydrobiol.

