# Supplementary material for: Benthic macroinvertebrates as reference indicators for monitoring of anthropogenic isotope 137Cs contamination in the marine environment
Source: Environ Sci Pollut Res Int. 2021 Oct 1;29(10):13822–34. doi: 10.1007/s11356-021-16538-y (PMC8810461; doi:10.1007/s11356-021-16538-y)
Supplement: Supplementary file 1 — (DOCX 34 kb) [file 11356_2021_16538_MOESM1_ESM.docx]

The biomass of macroinvertebrates in 2011 - 2018

| Year | | Limecola balthica | Mya arenaria | Astarte | Cerastoderma glaucum | Mytilus | Hydrobiidae | Hediste diversicolor | Marenzelleria | Saduria entomon |
| --- | --- | --- | --- | --- | --- | --- | --- | --- | --- | --- |
|  |  | Biomass [gdw m^-2^] | | | | | | | | |
| K6 | 2011 | 6.7 |  |  |  |  |  | 0.1 | 0.4 |  |
|  | 2012 | 4.3 | 0.4 |  |  |  |  | 0.3 | 1 |  |
|  | 2013 | 2 | 9.6 | 8.4 |  |  | 0.1 | 0.3 | 0.4 |  |
|  | 2014 | 1.5 |  |  |  |  |  | 0.3 | 0.4 |  |
|  | 2015 | 4.3 | 5.7 | 0.2 |  | 0.1 | 0.2 | 0.1 | 0.1 |  |
|  | 2016 | 24.8 | 1 | 18 |  | 0.1 | 0.2 | 0.7 | 1 |  |
|  | 2017 | 2.4 | 0.2 |  |  | 2.4 |  | 0.1 | 0.1 |  |
|  | 2018 | 12.5 | 2.7 | 0.9 |  | 12.5 |  | 0.6 | 0.5 |  |
| M3 | 2011 | 139.6 |  |  |  | 3.3 |  |  | 2.3 | 3 |
|  | 2012 | 26.6 | 10.4 |  |  |  |  |  | 3.4 | 1.3 |
|  | 2013 | 25.6 | 3.8 |  |  | 0.9 |  |  | 1.3 | 2 |
|  | 2014 | 21.6 | 2 |  |  |  |  |  | 1.2 | 0.6 |
|  | 2015 | 11.2 |  |  |  | 5 |  |  | 1.2 | 0.2 |
|  | 2016 | 9.8 | 8.5 |  |  |  |  |  | 0.8 |  |
|  | 2017 | 18 |  |  |  | 24.6 |  |  | 0.1 |  |
|  | 2018 | 16.3 | 2 |  |  | 16.4 |  |  | 0.2 |  |
| P104 | 2011 | 270.2 |  |  |  |  |  |  | 0.7 | 11.3 |
|  | 2012 | 191.8 |  |  |  |  | 0.1 |  | 1.8 | 5.1 |
|  | 2013 | 111.4 |  |  |  |  |  |  | 0.1 | 6.4 |
|  | 2014 | 81.7 |  |  |  |  |  |  |  | 4.7 |
|  | 2015 | 64 |  |  |  |  |  |  | 0.1 | 1.4 |
|  | 2016 | 46.2 |  |  |  |  |  |  | 0.2 | 1 |
|  | 2017 | 41.9 |  |  |  | 41.9 |  |  | 0.1 | 0.7 |
|  | 2018 | 17.8 |  |  |  | 17.8 |  |  |  | 1 |
| P110 | 2013 | 1.4 |  |  |  |  |  |  |  |  |
|  | 2015 |  |  |  |  |  |  |  |  |  |
|  | 2016 | 1.6 |  |  |  |  |  |  |  |  |
|  | 2017 | 5.4 |  |  |  | 5.4 |  |  |  |  |
|  | 2018 | 14.1 |  |  |  | 14.1 |  |  |  |  |
| P2 | 2014 |  |  | 119.1 |  | 2.5 |  |  |  | 0.8 |
|  | 2015 | 1.2 |  | 138.1 |  | 0.4 |  |  |  | 0.9 |
|  | 2016 | 0.1 |  | 58.5 |  | 0.2 |  |  |  | 0.6 |
|  | 2017 | 0.3 |  | 155.9 |  | 0.4 |  |  |  | 0.3 |
|  | 2018 |  |  | 65.9 |  | 0.1 |  |  |  | 0.4 |
| P3 | 2014 | 4.3 |  | 1.7 |  |  |  |  |  |  |
|  | 2015 | 5.3 |  | 3.2 |  |  |  |  |  |  |
|  | 2016 | 2.7 |  | 1.8 |  |  |  |  |  |  |
|  | 2017 | 5.7 |  | 7.7 |  | 5.7 |  |  |  |  |
|  | 2018 | 1.1 |  | 13 |  | 1.1 |  |  |  |  |
| ZN4 | 2012 | 65.7 |  |  |  |  |  |  | 0.3 | 1.9 |
|  | 2013 | 81.5 |  |  |  |  |  |  | 2.1 | 2 |
|  | 2014 | 66.9 | 0.5 |  |  |  |  |  | 0.9 | 0.9 |
|  | 2015 | 110.7 | 2.9 |  |  |  |  | 0.1 | 1.4 | 2.3 |
|  | 2016 | 74.2 |  |  |  |  |  |  | 1.7 | 0.9 |
|  | 2017 | 43 |  |  |  | 43 | 0.1 |  |  |  |
|  | 2018 | 47.4 |  |  |  | 47.4 |  |  | 0.4 | 1.1 |
| ZP6 | 2012 | 17.3 | 139.5 |  | 6 | 37.9 | 1.8 | 4.4 | 1.4 |  |
|  | 2013 | 22 | 156.7 |  | 4.4 | 0.2 | 5.8 | 4.1 | 0.9 |  |
|  | 2014 | 27 | 112.5 |  | 3.8 | 8.4 | 16.6 | 5.3 | 0.3 |  |
|  | 2015 | 1.7 | 13 |  | 0.3 | 3.7 | 4.2 | 0.3 | 0.1 |  |
|  | 2016 | 13.3 | 32 |  | 14.2 | 0.2 | 1.3 | 1.7 | 0.9 |  |
|  | 2017 | 3.4 | 19.8 |  | 1.2 | 11.4 | 1.1 | 1.8 | 0.2 |  |
|  | 2018 | 4 | 14.3 |  | 5.9 | 68.7 | 5.8 | 2.7 | 0.5 |  |
| B13 | 2011 | 32.3 | 286.3 |  | 6.8 | 76.9 | 4.9 | 8.3 | 4.8 |  |
|  | 2012 | 25.2 | 144.5 |  | 11.1 | 11.5 | 1.3 | 1.9 | 0.5 |  |
|  | 2013 | 21.9 | 103.9 |  | 10.2 | 1.4 | 5 | 2.1 | 0.3 |  |
|  | 2014 | 18.6 | 57.7 |  | 22.8 | 9.4 | 3.9 | 0.9 | 0.5 |  |
|  | 2015 | 9 | 72.9 |  | 10.8 | 6.4 | 6.1 | 1.4 | 0.8 |  |
|  | 2016 | 15.7 | 61.8 |  | 6.6 | 0.5 | 3.2 | 0.5 | 0.6 |  |
|  | 2017 | 12.2 | 31.8 |  | 1.5 | 21.2 | 0.9 | 0.4 | 0.6 |  |
|  | 2018 | 12.2 | 65.5 |  | 9.6 | 28.9 | 1 | 0.6 | 1.1 |  |
| L7 | 2011 | 11.8 | 8.3 |  |  |  |  | 0.4 | 2.5 |  |
|  | 2012 | 12.7 | 8.9 |  | 1.8 |  |  | 0.8 | 0.8 |  |
|  | 2013 | 4.9 |  |  | 1 |  | 0.1 | 0.5 | 0.6 |  |
|  | 2014 | 3.9 |  |  |  |  | 0.3 | 0.3 | 0.9 |  |
|  | 2015 | 19.8 | 11.3 |  |  |  | 0.4 | 1.4 | 1 |  |
|  | 2016 | 19.3 | 6.7 |  | 11 | 0.3 | 1.5 | 1.8 | 1.2 |  |
|  | 2017 | 1 | 31.2 |  |  | 1 | 0.1 | 0.3 | 0.5 |  |
|  | 2018 | 13.4 | 1.3 |  | 0.5 | 13.4 | 0.1 | 0.6 | 1.2 |  |
| P16 | 2011 | 1.3 | 1.3 |  | 2.9 |  | 3.4 | 0.3 | 1.5 |  |
|  | 2012 | 19.2 | 89.9 |  | 0.9 |  | 0.2 | 1.6 | 0.8 |  |
|  | 2013 | 3.1 | 12.5 |  | 1.4 |  | 0.5 | 0.4 | 0.4 |  |
|  | 2014 | 3.3 | 16.9 |  | 1.3 |  | 0.1 | 0.3 | 1.1 |  |
|  | 2015 | 20.7 | 56.5 |  | 15.5 | 0.2 | 1.3 | 0.8 | 0.8 |  |
|  | 2016 | 0.8 | 0.8 |  | 4.5 |  | 0.3 | 0.3 | 1.8 |  |
|  | 2017 | 15.2 | 25.9 |  | 2.1 | 15.3 | 0.4 | 0.3 | 0.5 |  |
|  | 2018 | 2.3 | 59.4 |  | 21.9 | 43.4 | 1.5 | 0.8 | 1.4 |  |
| Z | 2011 | 46.5 | 0.3 |  | 1.1 |  | 0.8 | 1.6 | 1.8 |  |
|  | 2012 | 27.8 | 11 |  | 31.4 |  | 1.3 | 1.2 | 0.9 |  |
|  | 2013 | 33.2 | 12.8 |  | 41.4 |  | 0.7 | 0.5 | 0.8 |  |
|  | 2014 | 21.8 | 8.8 |  | 3.8 |  | 0.1 | 0.8 | 0.6 |  |
|  | 2015 | 63.2 | 6.4 |  | 19.5 |  | 1.4 | 0.9 | 1.1 |  |
|  | 2016 | 34.7 | 12.9 |  | 52.7 | 0.4 | 1.7 | 0.8 | 0.9 |  |
|  | 2017 | 17.4 | 3 |  | 24.5 | 17.4 | 0.7 | 0.4 | 0.6 |  |
|  | 2018 | 17.7 | 1.9 |  | 9.8 | 17.7 | 0.1 | 0.8 | 1.1 |  |
| P140 | 2011 | 0.1 |  |  |  |  |  |  |  |  |
|  | 2017 | 0.1 |  |  |  | 0.1 |  |  |  |  |
| P5 | 2011 |  |  |  |  | 0.2 |  |  |  |  |
